# Sympathetic System in Wound Healing: Multistage Control in Normal and Diabetic Skin

**DOI:** 10.3390/ijms24032045

**Published:** 2023-01-20

**Authors:** Evgenii Ivanov, Marina Akhmetshina, Aleksei Erdiakov, Svetlana Gavrilova

**Affiliations:** Faculty of Medicine, Lomonosov Moscow State University, 119991 Moscow, Russia

**Keywords:** diabetes mellitus, wound healing, sympathetic system, norepinephrine, acetylcholine, NPY, VIP, NGF, keratinocytes, angiogenesis

## Abstract

In this review, we discuss sympathetic regulation in normal and diabetic wound healing. Experimental denervation studies have confirmed that sympathetic nerve endings in skin have an important and complex role in wound healing. Vasoconstrictor neurons secrete norepinephrine (NE) and neuropeptide Y (NPY). Both mediators decrease blood flow and interact with inflammatory cells and keratinocytes. NE acts in an ambiguous way depending on receptor type. Beta2-adrenoceptors could be activated near sympathetic endings; they suppress inflammation and re-epithelialization. Alpha1- and alpha2-adrenoceptors induce inflammation and activate keratinocytes. Sudomotor neurons secrete acetylcholine (ACh) and vasoactive intestinal peptide (VIP). Both induce vasodilatation, angiogenesis, inflammation, keratinocytes proliferation and migration. In healthy skin, all effects are important for successful healing. In treatment of diabetic ulcers, mediator balance could be shifted in different ways. Beta2-adrenoceptors blockade and nicotinic ACh receptors activation are the most promising directions in treatment of diabetic ulcers with neuropathy, but they require further research.

## 1. Introduction

Diabetes mellitus is associated with several common comorbidities. Diabetic neuropathy is very common among patients with severe disease. Up to 50% of patients experience diabetic neuropathy at some point in their life. In addition to direct clinical manifestations, neuropathy contributes to the development of other complications of diabetes. Among other complications, diabetic neuropathy increases foot ulcer risk to 25% [1].

Diabetic ulcers are severe chronic wounds that often occur on the distal parts of lower extremities. They are resistant to treatment and contribute to the majority of nontraumatic leg amputations [2,3,4]. Some surgical and orthopedic treatment tactics improved diabetic ulcer healing rates [5,6]. The new generation of glycaemia-controlling medications has also alleviated the burden of diabetic ulcers. Despite these advances, these treatments are often not enough to improve patients’ conditions [7,8]. There is an urgent need for new pathogenetic ways to treat diabetic ulcers. Diabetic wound pathogenesis involves almost all major diabetic pathways and complications. Largely, wound progression is correlated with skin ischemia. Clinically, wounds with reduced skin blood flow are called angiopathic [9,10]. Some severe ulcers have a weak correlation with diabetic angiopathy. This subset of diabetic ulcers is thought to be completely neuropathic [11,12,13]. One of the most prominent clinical forms of neuropathic wound is Charcot neuroarthropathy [12,14]. But neuropathic factors can reveal themselves in different clinical forms. In any diabetic wound, both local ischemia and neuropathy are important, but in different proportions. Major surgical interventions and some medications were designed to restore microcirculation in wound area, yet they are less effective in neuropathic wounds [11,15]. Also, there are several important intermediate factors, like excessive inflammation, reactive oxygen species hyperproduction, metabolic disturbances and epigenetic changes [16,17,18,19].

Different components of diabetic ulcer pathogenesis have received unequal attention. There are more than 5000 reviews about diabetic ulcers in Medline database. Many of those are related to therapy options, wound infections, pathological inflammation, and microcirculation. Among them we have found nine reviews dedicated to neuropathy’s involvement in diabetic wound healing. The oldest one lists some general ways by which neuropathy could affect diabetic ulcers without pathogenetic mechanisms [20]. One review summarizes information about neurovascular control neuropathic impairments [21]. Five works give a detailed picture of neuropeptide and neurotrophic factors’ involvement in diabetic wound healing [22,23,24,25,26]. S. Sun et al. in a recent review focused β-adrenergic involvement and β-blockers treatment, though they did not touch a-adrenoceptors and cholinergic fibers [27]. In the most recent work, N.C. Nowak et al. gave a comprehensive review for sensory fibers’ involvement and neuropeptides. We unequivocally recommend this paper as the most interesting and complete picture of neuropathy in diabetic wound healing [28]. There are also a few reviews about nervous system involvement in normal wound healing or in different pathologies. L. Pan et al. described sympathetic interaction with angiogenesis in detail. Subsequently, we will give less credit to this aspect of autonomic nervous system role, but for more information, we recommend this article [29]. M. Ashrafi et al. and D. Gupta et al. mostly describe neuropeptide functions [30,31]. Important information was collected in reviews of intraepidermal noradrenaline and acetylcholine synthesis. They are complementary to our work, but either do not have information about wound healing and diabetes or do not make clear distinctions between nerve-derived and keratinocytes-derived neurotransmitters [32,33,34]. There is much information in the literature about the general sympathetic nervous system’s role as inflammation modulator. While those articles do not cover wound healing, they provide valuable concepts of adrenergic inflammation control. Additional to our work, information is reviewed by G. Pongratz and R. Straub [35].

Overall, the autonomic nervous system’s role in wound healing in normal and diabetic wounds has not been completely explained in all the current reviews. It is not clear how adrenergic, cholinergic and neuropeptide regulation mechanisms are integrated during the wound healing process. At the same time, there are many fragmentary studies and much broad evidence that these mechanisms are important. Autonomic diabetic neuropathy affects more than 20% of patients with diabetes [36]. Cardiovascular diabetic neuropathy is diagnosed most frequently, but studies provide clues that diabetes affects all parts of the autonomic nervous system [37]. While cardiac autonomic neuropathy is present in 43–66% of patients with diabetic wounds, other forms of autonomic neuropathy are probably prevalent as well [38]. Beta-blockers underwent some trials in diabetic ulcers, but we could better target the autonomic nervous system with a clearer view of underlying mechanisms. Neuropeptides’ involvement and angiogenesis control were thoroughly reviewed, and we will mention them briefly. We focused on articles about norepinephrine, acetylcholine, related nerve fibers and their interactions with effector cells on different steps of the healing process. Our aims are to compare known mechanisms with denervation studies results and clinical data, and to find important empty spaces in the current state of knowledge.

## 2. Methods

We conducted a literature (narrative) review; therefore, strict methodological criteria were not applicable. The search was performed in several steps for different parts of review in Medline, Scopus, and Web of Science databases. Below, we give search queries in Medline format, total results without duplicates through Medline database and final addition from all databases minus results from previous queries. Preclinical and clinical articles with any relevant disease model or disease were picked for analysis. Any article type with full text available was analyzed, and non-English works were auto-translated to extract their main positions. The search was conducted between 10 November 2022 and 10 December 2022.

(1) (“wound healing” [Mesh] OR skin wound* OR diabet* wound* OR diabet* ulcer*) AND (“Autonomic Nervous System” [Mesh] OR autonom* nerv*)—458 results, 78 to analysis
(2) (“wound healing” [Mesh] OR skin wound* OR diabet* wound* OR diabet* ulcer*) AND (“Receptors, Adrenergic” [Mesh])—59 results, 32 to analysis
(3) (“wound healing” [Mesh] OR skin wound* OR diabet* wound* OR diabet* ulcer*) AND (“Epinephrine” [Mesh] OR “Norepinephrine” [Mesh] OR epinephrine OR norepinephrine)—365 results, 61 to analysis
(4) (“wound healing” [Mesh] OR skin wound* OR diabet* wound* OR diabet* ulcer*) AND (“Receptors, Cholinergic” [Mesh])—41 results, 18 to analysis
(5) (“wound healing” [Mesh] OR skin wound* OR diabet* wound* OR diabet* ulcer*) AND (“Acetylcholine” [Mesh])—50 results, 2 to analysis
(6) (“wound healing” [Mesh] OR skin wound* OR diabet* wound* OR diabet* ulcer*) AND (“Neuropeptide Y” [Mesh] OR NPY)—33 results, 5 to analysis
(7) (“wound healing” [Mesh] OR skin wound* OR diabet* wound* OR diabet* ulcer*) AND (“Vasoactive Intestinal Peptide” [Mesh] OR VIP)—257 results, 7 to analysis

## 3. Sympathetic Neurotransmitters: Neuronal and Paracrine Sources

### 3.1. Sympathetic Regulation in Normal Skin

**Figure 1:**
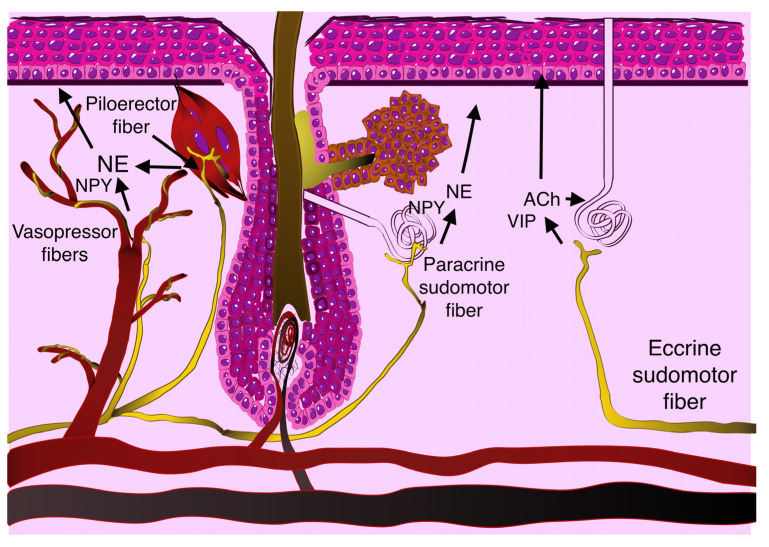
Normal autonomic skin innervation.

Autonomic nerves lie in dermis near blood and lymphatic vessels and dermal appendages and except for facial skin are only sympathetic. These fibers belong to cutaneous vasoconstrictor neurons, piloerector neurons and sudomotor neurons (Figure 1) [39]. Both types have C-type unmyelinated axons and are located in paravertebral ganglia [40]. Microneurographic studies have shown that one sympathetic unit innervates 24–350 mm^2^ of skin [41]. Immunolabeling histological studies allowed the differentiation of noradrenergic sympathetic fibers near arterioles or erector pili muscles and apocrine sweat glands and cholinergic sympathetic fibers near eccrine sweat glands [42]. These fibers lay in dermis at different depths. Contrary to sensory fibers, sympathetic fibers do not directly penetrate epithelium basal membrane [43].

Vasoconstrictor terminals innervate every arteriole in the skin; therefore, they are rather equally distributed. Some studies have also found their divisions alongside capillaries, where they could change endothelial integrity and conduct retrograde signals. In normal skin, vasomotor sympathetic fibers reduce blood flow. Other adrenergic fibers activate piloerection and regulate hair follicle cells’ activity. Also, some fibers innervate veins and collect lymphatic ducts, though their innervation is scarce. All adrenergic nerve endings primarily release epinephrine and neuropeptide Y (NPY), also different POMK—derived peptides. Sudomotor fibers increase sweating in stress reactions and heat dissipation. Cholinergic sudomotor neurons among acetylcholine release vasoactive intestinal peptide (VIP) [44]. Further we will see that sympathetic fibers have additional roles in wound resolving.

Deep soft tissue wounds can involve subcutaneous adipose tissue and superficial muscles. Adipose tissue has dense sympathetic innervation that directly regulates energetic balance and heat production [45,46]. Skeletal muscles contain only sympathetic fibers that innervate blood vessels, though recent evidence indicates their role in modulation of muscle contraction force [47,48]. We lack data on possible involvement of adipose and skeletal muscle sympathetic fibers in soft tissue wound healing. Therefore, we will further discuss dermal sympathetic fiber role and general sympathetic activation or denervation.

### 3.2. Neurotransmitter Synthesis in Skin Cells

Resident skin cells regulate normal tissue properties by paracrine release of signaling molecules. Among other, they produce catecholamines and neuropeptides and present different cell surface receptors. Keratinocytes express enzymes for norepinephrine (NE) and acetylcholine (ACh) synthesis and various receptors for them through all epidermis layers [33,49]. Keratinocytes vesicles contain tyrosine hydroxylase and phenylethanolamine-*N*-methyl transferase (PNMT), norepinephrine synthesis enzymes. Norepinephrine was also found in an isolated keratinocytes culture media [50]. In normal skin, the most immature basal keratinocytes produce more norepinephrine. NE increases calcium influx in the nearest cells through β_2_-AR, thus promoting keratinocyte maturation. Beta-adrenoreceptors are the best described in skin. They were found on keratinocytes from different skin localizations and on fibroblasts. Epidermis is densely labeled for β_2_-adrenoceptors in all layers with no β_1_-AR presentation. Sweat glands also have high β_2_-AR presentation; sebaceous glands are not labeled [51].

Therefore, it is proposed that intrinsic skin NE release is crucial to the native epidermis architecture. Several diseases, like atopic dermatitis and psoriasis, both impair keratinocytes differentiation and decrease norepinephrine levels [52].

Almost all human cells produce acetylcholine to a certain degree; therefore, active ACh depends on synthesis to acetylcholinesterase (AChE) hydrolysis ratio. All viable keratinocytes have equal ACh synthetic end exocytosis capacity, but AChE activity is localized in basal layer. Therefore, in intact epidermis, ACh concentration increases from basal to upper layers. Keratinocytes express a wide variety of ACh receptor genes—different non-muscular nAChR subunits and M_1_–M_5_ mAChR subtypes. The acetylcholine receptors are unequally distributed through the epidermis, and different acetylcholine actions in the different maturation stages have been speculated [35]. In the literature, a_3_, a_5_, b_2_, b_4_ nAChR subunits were detected in the epidermal basal layer and lower stratum granulosum, a_9_ nAChR subunits—in the basal layer and in the lowest suprabasal epidermis, a_7_, a_10_ and b_1_ nAChR subunits—in the upper stratum spinosum and stratum granulosum. In addition, M_1_ and M_4_ muscarinic acetylcholine receptors were described in the suprabasal layers, M_2_, M_3_ and M_5_—in the lower layers [53]. While different receptor subtypes have opposing effects, keratinocytes could respond to ACh differently dependent on maturation stage. Overall, normal ACh stimulation in intact epidermis promotes keratinocyte maturation through different phenotypes. Also, ACh is important for steady sebaceous and sweat gland secretion.

Catecholamines and acetylcholine regulation of skin homeostasis can be explained by both in-place paracrine regulation and neurogenic regulation [54]. We need more evidence to evaluate effects from two sources separately. As some authors propose, the high rate of neurotransmitter metabolism in basal keratinocytes is important to separate the two regulatory compartments. NE, ACh from sympathetic fibers could freely interact with skin appendages, basal epidermis layer and dermal cells. Because of uptake by basal keratinocytes, neuronal transmitters have a little effect on upper epidermis layers and vice versa. But in soft tissue wounds, natural barriers are compromised. All cells that migrate into the wound area are equally exposed to neurotransmitters and their regulation is important.

## 4. Sympathetic Regulation in Normal Wound Healing

Soft tissue healing, like any acute inflammation, has several stages: vessel dilatation and permeabilization, proliferation and reparation [55]. The autonomic nervous system regulates all stages of wound healing in the wound area, in intact skin near the wound and in the whole body. Sympathetic mediators could constrict arteries in the skin to prevent excessive blood loss. In intact skin around the wound, sympathetic fibers are crucial in maintaining physical properties of the skin. Without normal regulation, sweating skin can become dry and vulnerable to infections. In the wound area catecholamines, acetylcholine and neuropeptides modulate leukocyte activity, reepithelization, wound contraction, and other important processes.

### 4.1. Inflammation and Immune Cells

In the first days after wounding, acute inflammation decides the fate of the healing process. In minutes after initial damage injured cells, resident immune cells and tissue debris affect local blood vessels and attract granulocytes from blood stream. Vasodilatation and increased vessel permeability enhance leukocyte transcytosis. Sympathetic fibers modulate acute inflammation in several ways with different neurotransmitters and receptors.

As any serious trauma usually acts as a stressor, the catecholamine level in blood quickly increases, simultaneous with local sympathetic fibers’ discharge. Catecholamines increase skin arterioles’ resistance, shunting more blood to vital organs. This effect could delay leukocyte migration, but it also prevents excessive blood loss. Some older works have stated that catecholamines also increase capillary permeability [56,57]. Later research contradicted this finding. Lack of sympathetic activation, for example in diabetic skin, rather increased capillary leak through lower vessel tone [58,59]. While most cytokines and inflammation mediators in the wound area increased capillary permeability, catecholamines act as antagonists.

Catecholamines effectively modulate leukocytes’ activity [60]. Adrenoceptors have been found on all types of leukocytes with different density, most abundant are β_2_-adrenergic receptors [61]. Information about other types of adrenergic receptors is controversial in different species and cell types. Next to β_2_ by quantity are a_1_, then a_2_ and β_1_-receptors, respectively [62]. a-adrenoceptors and β-adrenoceptors enhance opposite processes in leukocytes, but they have different affinity and abundance. β_2_ adrenergic regulation of immune response is the most studied and probably the most prevalent and important. β_2_-agonists decrease TNFa and other pro-inflammatory cytokines release, leukocyte migration and chemotaxis [63]. IL-10 concentration is elevated rapidly after β_2_-AR activation leading to immunosuppression or localized inflammation [64]. Possible mechanisms include protein kinase A (PKA)—mediated NF-kB suppression and β-arrestin 2 protein synthesis [64,65]. A. Gosain et al. studied norepinephrine effects on activated neutrophils and macrophages, isolated from rat wounds. Both types of cells have shown significantly reduced phagocytic activity by PKA activation. Macrophages were inhibited both by physiological and pharmacological NE doses, while neutrophils were inhibited only by pharmacological NE dose [66,67].

Less data is available about a-adrenoceptor’s role in soft tissue wound healing. In most cases, they have shown pro-inflammatory properties. a-adrenergic agonists increase pro-inflammatory cytokines production, immune progenitor proliferation and reactive oxygen species production [68,69]. In other inflammatory situations a-adrenoceptors stimulation increases many pathological reactions. In systemic inflammation, a_1_-adrenergic stimulation increases the release of TLR-driven cytokines from macrophages [70]. a_1b_ receptors also have shown ability to form heterodimeric complexes with chemokine receptors and regulate their activity [71,72].

Overall, acute catecholamines release reduce inflammation through β_2_-adrenergic receptors. This is interesting, because inflammation is vital in normal wound healing [73,74]. Despite thousands of works it is still impossible to predict when inflammation becomes deleterious or when anti-inflammatory agents become harmful. For example, glucocorticoids have been shown to prevent wound healing or even to induce wounding in different situations [75,76]. On the other hand, in some wound models, glucocorticoids improve healing [77]. Similarly, β-AR affecting drugs are very inconsistent in different inflammation models and diseases [78,79,80,81,82,83,84,85,86]. In simple wounding model cell proliferation rate, neutrophiles and mast cells migration, myofibroblast density and the blood vessels volume density were increased by a beta-blocker, while healing was delayed overall [87]. To the contrary, after propranolol administration in streptozotocin-induced rat diabetes model, the wound area was smaller 7 and 14 days after wounding in propranolol group, and inflammatory cells number and MMP-9 level were reduced [88].

Therefore, we should study pro- and anti-inflammatory agents in more detail to find finer switching mechanisms. Because β_2_-AR related effects are dominant, Pongratz et al. hypothesize that sympathetic system nerve endings prevent deleterious inflammation spreading and tissue damage. β-adrenergic receptors bind NE with lower affinity, than a-adrenergic receptors. Sympathetic nerve terminals release NE, and in proximity it binds with both β- and a-adrenergic receptors on leukocytes with subsequent β_2_-AR induced IL-10 release. Farther from NE source, a high-affinity a-adrenergic receptor binds more mediator, than β-adrenergic receptors and TNFa release prevails over IL-10 production. Therefore, intact sympathetic fibers reduce inflammatory response in intact wound margins and increase closer to the wound’s center. Possible repulsion of sympathetic fibers from the inflammation area would also positively modulate inflammation [35]. This concept only includes catecholamines, though other sympathetic transmitters in the skin also could be important for net inflammation balance.

As sudomotor sympathetic nerves release acetylcholine, they could trigger acetylcholine responses aside from vasodilation. As ubiquitous paracrine agent, acetylcholine is an important immune system mediator. Acetylcholine has been revealed as a pro-inflammatory mediator in many studies [89,90]. Leukocytes produce different types of cholinergic receptors: M_1_–M_5_ muscarinic receptors, a_3_, a_5_, a_7_, a_9_, a_10_ nAChR subunits [91,92]. Both cholinergic receptor types inhibit cytokine secretion from leukocytes, though mAChR are better-researched [93,94,95]. Considering that, acetylcholine release also could decrease inflammation in wound area. Contrary to catecholamines, ACh promotes vasodilation that stimulates leukocyte extravasation [53,96].

Vasoactive intestinal peptide (VIP) is characteristic for cholinergic neurons, including sympathetic sudomotor neurons. In the acute phase of inflammation, VIP induces histamine and bradykinin release from mast cells. Therefore, VIP itself and vasoactive inflammatory mediators induced by its actions promote vasodilatation in wound margins. In later stages, VIP could realize its anti-inflammatory properties. In different circumstances VIP could increase Treg cells level and protect them from apoptosis, inhibit TNFa and IL-6 secretion [97,98,99].

Neuropeptide Y (NPY) is produced in skin primarily by vasomotor sympathetic nerve endings. Its role in soft tissue wound healing is not completely understood. In other pathologies NPY acts as pro-inflammatory agent [100,101,102,103]. NPY induces cytokine production in leukocytes through Y1 and Y5 receptors, but other types of NPY receptors with other roles also were scarcely described [102,104].

Among typical skin sympathetic neurotransmitters, only NPY can possibly act as a pro-inflammatory without immunosuppressive functions in normal wound healing. To focus inflammation near the wound’s margins immune cells need to repel nearby sympathetic fibers. Indeed, inflammatory sympathetic repulsion is a well-known phenomenon. A special class of semaphorin molecules called nerve repellent factors regulates neurite outgrowth. They include semaphorin 3F(SEMA3F), plexin-A2, neuropilin-2 and other factors [105]. In inflamed tissues, including diabetic Charcot foot, semaphorin 3C is highly expressed with lower sympathetic fibers’ density [106]. S. Clatt et al. tested whether cytokines and hormonal factors released in inflamed tissue also have repellent properties. TNF-α repelled nerve fibers with moderate to strong effects (0–100%). High concentrations of dopamine and norepinephrine (10^−6^ M) induced weak but significant nerve fiber repulsion (up to 20%). Stimulation with low concentrations of 17β-estradiol (10^−10^ M, but not 10^−8^ M) repelled SNFs [107]. Systemic inflammatory responses in sepsis also induced nerve repulsion in primary immune organs. D. Hoover et al. have found that in patients with sepsis there are about 16% of normal sympathetic fibers. While spleen innervation provides ambiguous immune regulation, it’s dysfunction could both decrease inflammation specificity and increase detrimental effects [108]. Major aspects of sympathetic neurotransmitters’ interaction with immune cells were placed in Table 1. 

### 4.2. Keratinocytes

Keratinocytes (KC) are activated after wounding among other cells through the first minutes and hours. Keratinocytes are characterized by keratin expression profile, which defines the KC phenotype. In healthy skin there are basal KC, that proliferate and replenish lost corneocytes through different maturation stages. After wounding basal KC and some mature KC switch their phenotype to activated or contractible. Activated KC can migrate, proliferate and are crucial for re-epithelialization. Contractible KC pull the extracellular matrix to make wound area smaller. Without any kind of epidermis, the wound has a great risk of becoming infected or chronic [109,110].

Because intact epidermis cells synthetize NE, they could affect each other and prevent inflammatory activation. In vitro β_2_-adrenergic receptors activation inhibits keratinocyte migration [111,112]. Epinephrine is a more potent keratinocyte migration inhibitor than norepinephrine [113]. Cellular events after keratinocytes β_2_-AR activation include serine/threonine phosphatase PP2A activation, extracellular signal-related kinase (ERK) dephosphorylation and promigratory signaling cascade blockade [114]. Keratinocyte proliferation is also inhibited by β_2_-AR agonist isoproterenol [115].

Unlike in immune cells, keratinocytes β_2_-AR contribute to pro-inflammatory cellular response as well. Epinephrine increases interleukin production, and β_2_-AR—TLR crosstalk significantly augments inflammatory response [116]. Because epidermis is always in contact with the outer world and its germs, cytokines could be important in maintaining skin defenses. While β_2_-AR blocks KC activation, an increased cytokines level possibly could overcome this effect [117]. For now, this question remains open.

Wound modeling in keratinocytes culture leads to rapid norepinephrine release and persistent downregulation of β_2_-AR protein and catecholamine synthesis enzymes gene expression [118]. Keratinocyte culture scratch wounding downregulated expression of β-adrenoceptors genes, tyrosine hydroxylase and PNMT genes. While β_2_-adrenoceptors functional activity remained depressed, their gene expression returned to the baseline. With decreased β_2_-AR stimulation, keratinocytes produced more norepinephrine, which impaired their migration activity in wound edges. Effects were diminished by β_2_-AR selective antagonist ICI-118,551, β_1_-AR selective antagonist bisoprolol did not change them [118]. R. Sivamani et al. in vitro and in mouse burn model have found detrimental role of β_2_-AR after epinephrine exposure. In vitro keratinocytes blockade was achieved by physiological stress-induced epinephrine concentrations [119].

As with immune cells, alpha-adrenoceptors have opposite effects after KC activation. In studies, a_2A_/a_2C_-adrenoceptors knockout transgenic mice have shown accelerated wound contraction and re-epithelialization. On the other hand, a_2_-adrenoceptors on the presynaptic membrane reduce catecholamine release, therefore external a_2_-AR sympathetic activation could improve wound healing through inhibited NE release and lower β_2_-AR activation [120]. In vitro a_2_-ARs increase keratinocyte migration. Under low norepinephrine concentration a_2_-ARs overcome β_2_-adrenoceptors and their stimulation induces rapid migration [121]. Probably, a-adrenoceptors activation prevents KC from switching back to a stable basal or mature phenotype.

Acetylcholine also is abundant in epidermis and can act on the keratinocytes directly via cell receptors. Muscarinic receptors of five molecular subtypes and nicotinic receptors were found in keratinocytes and melanocytes [33]. Combined acetylcholine receptors blockade in vitro leads to the complete organotypic skin culture growth and proliferation inhibition. The nAChR receptors blockage led to less prominent changes than did the mAChR blockage in terms of culture thickness and maturation marker genes’ expression [122,123]. Important acetylcholine function is a keratinocytes cohesion stimulation [53,124]. Like NE, ACh also increases cytokine synthesis in keratinocytes. We propose that, by the same logic, cytokine stimulation can compensate direct inhibition [125]. In some reports M_3_ mAChR activation inhibits KC migration, while M_4_ mAChR activates migration [54]. In vitro experiments establish that a_9_ nAChR is important for migration start; without it KC remained attached to the surface. A huge number of receptors with opposite effects reduced the chances to successfully target cholinergic system in wound healing [34,126]. Neuropeptides’ interactions with KC are less researched. VIP induces keratinocytes migration and proliferation, and probably it is one of the most promising targets for study [127,128,129]. NPY receptors Y1 and Y4 were detected in all epidermal layers of the human skin [130]. Interestingly, while CGRP and VIP activate cAMP in keratinocytes culture, leading to increased cell proliferation, NPY downregulates cAMP with the opposite effects. It is probable that NPY blockers also could have some useful implications [131]. Major aspects of sympathetic neurotransmitters’ interaction with keratinocytes were placed in Table 2.

### 4.3. Fibroblasts

In later stages of the wound healing process, fibroblasts became key cellular elements. Their growth factors terminate inflammation, and their work defines the degree of scarring and functional restoration [132,133].

Fibroblasts produce almost all types of adrenergic and cholinergic receptors. In proliferation phase, β_2_-receptors become more beneficent, as they activate fibroblasts. In zebrafishes and porcine skin wound model β_2_-AR agonists inhibit contraction and fibrosis, reduce scar area, and improve scar quality [134]. β_2_-AR activates fibroblasts migration and attenuates cAMP-dependent matrix contraction [135,136]. After the beta-adrenoceptors blockade wound contraction and epidermal healing were delayed, decreased hydroxyproline levels, collagen density and neo-epidermal thickness were in evidence [87]. After propranolol administration in streptozotocin-induced rat diabetes model, the wound area was smaller 7 and 14 days after wounding in propranolol group; MMP-9 level was reduced and cell proliferation, mast cells number, collagen deposition, blood vessels density and nitric oxide levels were increased [88]. In Pullar et al. work, β_2_-AR antagonism increased angiogenesis, fibroblast functions, re-epithelialization [137]. Therefore, real data is inconsistent and more high-quality research is needed.

Less data is available about alpha-adrenoceptors, but in most cases, they have shown pro-inflammatory properties. Alpha-adrenergic agonists increase pro-inflammatory cytokines production, immune progenitor proliferation and reactive oxygen species production, as well as TGFb synthesis [68,69]. Dermal fibroblasts also express several acetylcholine receptors: a_3_b_2_, a_5_, a_7_, a_9_ nAChRs, M_2_, M_4_, and M_5_ mAChRs [53,124]. Cellular effects were not properly studied, but mostly, acetylcholine receptors activation promotes matrix formation or remodeling [138].

### 4.4. Blood Vessel Cells

Finally, fast, and functional restoration requires increased blood supply. All sympathetic neurotransmitters and neuropeptides affect angiogenesis. As this topic is described in many reviews, we briefly discuss several points [9,29,139,140,141,142].

Both in murine wound models and in human skin wounds, β_2_-AR activation prevents phospho-ERK cytoskeleton remodeling and delays wound re-epithelialization and healing [143]. Interestingly, the same effects could be seen in vascular smooth muscle cell culture [144,145]. β_2_-AR activation also decreases angiogenesis, and endothelial cells’ migration via cAMP-dependent mechanisms [146]. It is possible that a_1_-AR gene overexpression in vascular cells could lead to altered circulatory dynamics. Indirectly, these alterations could contribute to dysfunctional keloid scars that maintain high a_1_-AR production [147].

High SNS activity leads to stable NPY increase, hence vascular tone is permanently elevated, like in arterial hypertension. Diabetes mellitus is often accompanied by lower NPY production in skin [148]. NPY released by sympathetic nerve fibers stimulates endothelial cells proliferation and migration [149,150]. NPY Y2 receptors’ deletion in mice delays the wound healing by an angiogenesis blockade [148].

## 5. Wound Healing in Denervation Models

Experiments with denervated animals revealed the significant role of nerve endings in wound healing, though results are not always consistent. Partial surgical denervation delays wound healing in some studies and does not affect healing process in others. Complete denervation worsens skin regeneration in almost all studies [23]. As an exception, Ranne et al. did not find histological changes in rat groin skin after surgical denervation, though there could be functional impairments [150]. Further we will compare denervation experiments with information about different parts of sympathetic interactions with wounds.

Peripheral sympathetic denervation is executed with high dose 6-hydroxydopamine (6-OHDA). K. Saburo et al. described morphological changes in rat burns healing after 6-OHDA sympathectomy. There were fewer capillaries in granulation tissue, vessels were dilated, and the collagen was fine fibrous, rather than the thick bundles of the control group [151]. Probably, these results go in line with NE deficiency. L. Kim et al. reported decreased linear skin incisions healing in rats after 6-OHDA sympathectomy (two-times more rats in the control group were healed by day 14). Functional sympathetic blockade in rats with propranolol (beta-blocker) and phentolamine (alpha-blocker) reduced wound contraction and re-epithelialization but increased total cell proliferation, possibly by inflammatory cells [152]. It is interesting because catecholamine receptor blockade should prevent KC inhibition. Probably, as we mentioned before, NE, importantly, increases cytokine production. In previous work authors have found that general sympathetic denervation with 6-OHDA accelerates wound contraction in rats but delays epidermal restoration [153]. Z. Zheng et al. in similar experiment confirmed increased wound contraction and delayed reepithelization and have shown decreased levels of norepinephrine, epidermal growth factor, IL1-beta, NG2 proteoglycan and desmin [154]. Interestingly, A. Jurjus et al. reported other results in rat burn model with guanethidine denervation, that induce postganglionic neurons depletion. Wound surface area was reduced faster after sympathectomy, contrary to decreased healing rate in 6-OHDA models [155]. One experiment has shown that exogenous local SNS activation with low doses of 6-OHDA increased epidermal wound healing by 35% and dermal strength was increased by 43% [156].

Autonomic response could be predicted by comparing results of total and sympathetic denervation. Sensory denervation with capsaicin has a wide range of results [23]. In immature rat capsaicin treatment delayed cutaneous wound healing with increased proliferation and decreased apoptosis [157]. In some works, selective capsaicin sensory denervation had no impact on wound healing [158]. J. Wallengren et al. studied both capsaicin and surgical denervation wound healing models with negative results. With 70% nerve fibers depletion, the researchers found normal wound closure. Additionally, by day 10 they reported nerve fiber density recovery [159].

## 6. Sympathetic System in Diabetic Wounds Regeneration—Beneficial or Deleterious?

Wound healing is severely compromised in diabetes. There are multiple factors that decrease skin reparation capability in the diabetic condition. Diabetes mellitus is often accompanied by a diabetic neuropathy (DN). In the skin, DN leads to reduced nerve fiber density, neuropeptides deficiency and failed nerve regeneration. C. Chan et al. have shown that delayed wound healing in diabetic mice is related to 30–50% fewer axons at the wound margins, compared to 10–15% fewer axons in the skin before the wounding. Moreover, wound healing was accompanied by 70% adjacent hair follicles innervation reduction and worse axon plasticity [160]. D. Levy et al. described reduced CGRP+, SP+, VIP+ and NPY+ nerve fibers density in the skin of diabetic patients [161].

One of the major diabetic neuropathy forms is autonomic neuropathy. This could affect any part of the autonomic nervous system, though the most obvious clinical signs include dysmotility, urine incontinence and arrhythmias. The majority of thematic research has been focused on the sensory component of neuropathy in diabetic ulcer pathogenesis. But sensory neuropathy becomes prominent only after severe neuronal loss [162]. Despite normal basal line activity, stress tests could reveal the sympathetic system decrease much earlier. In type I diabetic subjects, exercises led to smaller and shorter norepinephrine and NPY elevation in blood [163]. Sensory neuropathy also could indirectly trigger altered sympathetic activity. Painful neuropathy is always accompanied by somatic changes, and mediated by sympathetic hyperactivation [164]. Also, while much information is transferred by C-type nerve fibers, both sympathetic and sensory nerve systems often suffer simultaneously. Perception and sympathetic skin response impairment are correlated in patients with diabetic polyneuropathies [165].

There could be different skin changes in diabetic patients, though in most cases all regulatory and trophic functions are reduced. Skin sympathetic reaction to intra-epidermal electrical stimulation correlates with diabetes severity, independent of clinical signs of neuropathy [166]. Sudomotor dysfunction is described as a high-sensitivity screening test to diagnose diabetes complications [167]. Diabetic neuropathy is accompanied by a_1_-AR gene overexpression in cutaneous vessels. Possibly, denervated structures overexpress a_2_-adrenoceptors and become more sensitive to other catecholamine sources [168]. Diabetes severely compromises microcirculation. Sympathetic fibers deficiency independently increases capillary leakage and reduces vasomotion. Indeed, the microcirculation state is dependent on both vessel wall thickness and local blood flow regulation [58].

Z. Zheng et al. studied the effects of chemical denervation with 6-OHDA in diabetic mice wounds. Sympathectomy was accompanied by smaller wound areas. To the contrary, histological regeneration scores were reduced in 6-OHDA group on day 21. Sympathectomy decreased the number of mast cells and norepinephrine, epidermal growth factor (EGF), interleukin-1 beta, NG2 proteoglycan, and desmin gene expression on the days 17 and 21t. Also, pericyte proliferation was reduced, which could explain vascular dysfunction. The authors concluded that 6-OHDA in diabetic mice delays re-epithelialization during wound healing by decreasing EGF but increases wound contraction by reducing IL-1β levels and the number of mast cells [154].

Diabetes is accompanied by a hyperinflammatory state. This is especially important in obesity-related conditions. White adipose tissue is a major source of inflammatory cytokines: IL-1, TNF-a, IL-6, chemokines etc. Dyslipidemia and insulin resistance have direct impacts on leukocytes and promote their activation. High glucose levels in blood also directly stimulate inflammation. Acute hyperglycemia causes acute IL-1a, IL-4 and IL-6 elevation. Even in healthy individuals, transient hyperglycemia induces major inflammatory transcription factor NF-kb gene expression. Reactive oxygen species are overproduced by leukocytes, and therefore many processes are compromised by oxidation [169,170].

Like other hyperinflammatory states, diabetes diminishes antimicrobial defenses. In the steady-state the immune system is always hyperactive, but its real defense power is low. Many studies report signs of pathological inflammation in diabetic ulcers [171,172,173]. Neutrophils have lower phagocytic activity index and intracellular killing activity. In early stages of wounding leukocyte infiltration could be decreased, but in chronic state neutrophiles and macrophages persist in wound margins. Finally, granulation tissue is produced slower and ulcer re-epithelialization is worse [174].

## 7. Targeting Sympathetic Nervous System in Diabetic Ulcers

SNS-targeting drugs were studied in several works to improve wound healing in different pathological conditions, but few of them were clinical trials. Timolol is one of the most extensively studied β-blockers for wound healing. H. Albrecht et al. studied combined thermal and radiation wounds in isolated human skin flaps. Beta-blocker timolol increased wound epithelialization by 5–20% [175].

The first clinical trials of systemic timolol administration refer to burn healing, though systemic effects in burns are distinct from regular wounds [176]. Topical timolol was studied in a couple of case studies with different nosology and in a few small clinical trials [177,178,179]. B. Thomas et al. conducted case-control study of topical timolol in chronic leg ulcers of diabetic or venous origin. They have found significant changes in mean wound area at 4, 8 and 12 weeks of complex treatment. Wound closure was almost two times as fast in the timolol group in all three checkpoints. Diabetic wounds and venous ulcer have shown similar response to treatment [180]. A. Ghanbarzamani et al. report positive results treating 64 patients’ graft sites after burn treatment [181]. T. Baltazard et al. treated 40 patients with venous leg ulcers with timolol gel or control. Twice as many patients in the timolol group demonstrated high ulcer area reduction [182]. For now, there are no completed clinical studies examining, with a clear design, timolol in diabetic wound healing. R. Caur et al. registered a phase two randomized clinical trial to test timolol in diabetic ulcers treatment. As of 2022, there are 30 patients enrolled, and the study could be completed in 2024 [183].

Topical muscarinic antagonists have shown preclinical effectiveness in reducing diabetic neuropathy symptoms, possibly through interaction with sympathetic nerve membranes. C.G. Jolival et al. reported results of 2% pirenzepine treatment in mice model of streptozotocin-induced diabetes. Topical delivery dose-dependently prevented tactile allodynia, thermal hypoalgesia and loss of epidermal nerve fibers. Drug withdrawal or frequency reduction reversed the effects, so muscarinic antagonists provide local symptomatic relief to diabetic neuropathy [184]. As far as we know, muscarinic antagonists were not tested in wound healing. There are few reports about nAChR agonists and other cholinergic agents in diabetic wounds healing. R.S. Dillon reported slower wound healing in six patients with diabetic neuropathy after atropine application and faster healing after methacholine (muscarinic agonist) application [185].

M. Kishibe et al. have studied topical nAChR agonists in diabetic mouse wounds. They have found diminished bacterial survival and systemic dissemination, as well as reduced wound TLR2 production. Reporting a_7_ nAChR impaired synthesis in human diabetic skin, authors proposed that a reduced cholinergic response accounted for the excessive inflammation [186]. M. Dong et al. have found that selective a_7_ nAChR agonist PNU282987 inhibits AGE-induced NF-kB activation and RAGE gene expression in diabetic wound macrophages. A reduced TNF-a level in diabetic mice was accompanied by accelerated healing rate, elevated fibroblast number and collagen deposition [187]. J-Y. Li et al. further discovered the same results in uncovered (non-diabetic) wounds. In wounds, covered with dressing, results were the opposite. nAChR7 activator inhibited re-epithelialization, angiogenesis and epithelial proliferation. Possibly, the effects are opposite because inflammatory processes are very different in covered and uncovered wounds [188]. Nicotine itself in low doses topically has shown promising results in accelerating healing of different wound types by angiogenesis promotion [140,189,190,191].

## 8. Conclusions

The sympathetic nervous system regulates skin homeostasis in different ways and at multiple levels. Therefore, we tried to classify the existing knowledge in this topic. We propose that there are three main ways SNS could interact with skin and wounds.

Firstly, vasomotor nerves suppress keratinocyte migration and proliferation, reducing inflammation by catecholamines through β_2_-adrenergic receptors. They are activated only by high norepinephrine concentrations; therefore, effects are prominent near vasoconstrictor nerve endings. NPY released near the vessels has similar effects and prevents inflammation and proliferation. Anti-inflammatory effects could be deleterious in the early stages of normal wound healing and skin cells in wound area could repel nerve endings. Intact skin vasoconstrictor nerve endings have a protective function and NPY also stimulates angiogenesis, which is important for distant wound margin. In the reparation phase, semaphorin concentration should fall, and regrowing nerve endings stimulate fibroblasts activity and matrix deposition through β_2_-AR.

Secondly, the same norepinephrine from distant nerve endings could stimulate cells in active wound centers through a_1_- and a_2_-adrenergic receptors. They display prominent pro-inflammatory properties and induce an activated keratinocytes phenotype. This pathway could be dangerous for patients with neuropathies, because a-adrenoceptors diminish blood flow and inhibit angiogenesis. Therefore, phase transition could be compromised and hyperinflammation could support chronic wounds. a-adrenoceptors also inhibit fibroblast activity and delay functional restoration.

The third way is sudomotor activation by acetylcholine and VIP. Simultaneously, they increase blood flow, induce angiogenesis, reduce inflammation, and stimulate keratinocytes migration and proliferation. Some of these effects are mixed by minor types of cholinergic receptors with opposite functions. Unfortunately, it is not clear when and where sudomotor neurons take part in wound healing. We can speculate that they could be beneficial in the proliferation and reparation phases. Probably, they are also repelled in acute inflammation phase and do not have distant mode of action.

With this concept we could predict severe healing dysregulation after sympathetic denervation. Lack of alpha-adrenergic stimulation will impair keratinocytes’ migration and decrease the healing rate. Early selective beta-adrenergic blockade could improve fast re-epithelialization but could be detrimental later. Experiments with sympathetic denervation generally lay in line with this hypothesis. Re-epithelialization is delayed, and the wound healing rate is lower, while inflammation is increased after 6-OHDA denervation. Functional beta-blockade impairs normal wound healing in some works, improves it in others, while alpha-blockade always improves it. Because results do not go in the same direction with 6-OHDA experiments, we propose that the sudomotor nerve endings effects are missing as a major positive contribution. In diabetic wounds beta blockade and cholinergic stimulation provide the best results in improving wound healing. We propose that diabetic keratinocytes lack stimulation to the point that inhibitory β_2_-adrenergic signals totally prevent re-epithelialization. While there are a few serious trials, we must be cautious in our interpretations and keep in mind that β_2_-effects are controversial in later stages of wound reparation. It is probable that experiments with VIP or NPY could give us more hope in the future.

## Figures and Tables

**Table 1:** Overview of sympathetic neurotransmitter interactions with immune cells.

Neurotransmitters	Norepinephrine/Epinephrine	Acetylcholine	VIP	NPY
Primary effect	Immunosuppressive	Immunosuppressive	Immunosuppressive	Pro-inflammatory
Source	Vasomotor fibers/keratinocytes	Sudomotor fibers/keratinocytes	Sudomotor fibers/keratinocytes	Vasomotor fibers/keratinocytes
Wound healing role—inflammation	Low concentration in wound area, high in healthy tissue around	Not clear	Not clear	Not clear
Primary receptor blockade	Switch to pro-inflammatory, better healing or hyperinflammation	Inflammation increase, better healing?	Inflammation increase, better healing?	Studied in some hyperinflammatory conditions only

**Table 2:** Overview of sympathetic neurotransmitter interactions with keratinocytes.

Neurotransmitters	Norepinephrine/Epinephrine	Acetylcholine	VIP	NPY
Primary effect	Inhibits activation, stimulates cytokines	Mixed through different receptors, stimulates cytokines	Activates KC	Inhibits activation
Source	Vasomotor fibers/keratinocytes	Sudomotor fibers/keratinocytes	Sudomotor fibers/keratinocytes	Vasomotor fibers/keratinocytes
Wound healing role—re-epithelialization	Stimulates KC to produce cytokines, probably to stop migration	Stimulates KC to produce cytokines, to start migration	Stimulates KC	Inhibits KC
Primary receptor blockade	Ambiguous data	Ambiguous data	Worse healing?	Better healing?

## Data Availability

Not applicable.
